# A Set of Eight Key Questions Helps to Classify Common Vestibular Disorders—Results From the DizzyReg Patient Registry

**DOI:** 10.3389/fneur.2021.670944

**Published:** 2021-04-29

**Authors:** Ralf Strobl, Michael Grözinger, Andreas Zwergal, Doreen Huppert, Filipp Filippopulos, Eva Grill

**Affiliations:** ^1^Institute for Medical Information Processing, Biometrics and Epidemiology, Ludwig-Maximilians-Universität München (LMU) Munich, Munich, Germany; ^2^German Center for Vertigo and Balance Disorders, University Hospital Munich, Ludwig-Maximilians-Universität München (LMU) Munich, Munich, Germany; ^3^Department of Neurology, University Hospital Munich, Ludwig-Maximilians-Universität München (LMU) Munich, Munich, Germany; ^4^Munich Centre of Health Sciences, Ludwig-Maximilians-Universität München (LMU) Munich, Munich, Germany

**Keywords:** vertigo, diagnosis, machine learning, surveys and questionnaires, clinical decision-making

## Abstract

Precise history taking is the key to develop a first assumption on the diagnosis of vestibular disorders. Particularly in the primary care setting, algorithms are needed, which are based on a small number of questions and variables only to guide appropriate diagnostic decisions. The aim of this study is to identify a set of such key variables that can be used for preliminary classification of the most common vestibular disorders. A four-step approach was implemented to achieve this aim: (1) we conducted an online expert survey to collect variables that are meaningful for medical history taking, (2) we used qualitative content analysis to structure these variables, (3) we identified matching variables of the patient registry of the German Center for Vertigo and Balance Disorders, and (4) we used classification trees to build a classification model based on these identified variables and to analyze if and how these variables contribute to the classification of common vestibular disorders. We included a total of 1,066 patients with seven common vestibular disorders (mean age of 51.1 years, SD = 15.3, 56% female). Functional dizziness was the most frequent diagnosis (32.5%), followed by vestibular migraine (20.2%) and Menière's disease (13.3%). Using classification trees, we identified eight key variables which can differentiate the seven vestibular disorders with an accuracy of almost 50%. The key questions comprised attack duration, rotational vertigo, hearing problems, turning in bed as a trigger, doing sport or heavy household chores as a trigger, age, having problems with walking in the dark, and vomiting. The presented algorithm showed a high-face validity and can be helpful for taking initial medical history in patients with vertigo and dizziness. Further research is required to evaluate if the identified algorithm can be applied in the primary care setting and to evaluate its external validity.

## Introduction

With a lifetime prevalence between 20 and 30% (1), vertigo and dizziness (VaD) belong to the most common complaints in primary care and emergency departments (2, 3). VaD have an annual prevalence of 9% in medical claim databases (4) and a high impact on daily life (5, 6).

In most cases, the diagnosis and treatment of VaD is straightforward (1, 7, 8). Inappropriate or delayed management of VaD, however, may contribute to chronic symptoms, increase disability, and cause secondary psychosomatic disorders (9–11).

While patients with VaD are mostly processed in primary care, a recent systematic review found considerable inconsistencies in the management of dizzy patients in this setting (12). Experts claim that taking a structured medical history is the key to make basic triage, namely, to decide if the patient can be managed in primary care, needs referral to a specialist, or, in the rare case of a life-threatening etiology of VaD (e.g., stroke), even needs emergency care. Concepts for a structured history taking such as the “Five Keys” (13) proposed that a limited number of symptom characteristics allows to successfully differentiate a majority of all VaD cases. Commonly recommended questions for rational history taking are whether VaD the complaints are paroxysmal or permanent, short or long lasting, spontaneous or triggered, of stereotypical presentation, direction-specific, or accompanied by neurological or otological symptoms (13, 14).

However, it has been argued that successful medical reasoning is often based on implicit experience and “gut feeling” that supplements explicit structured knowledge (15). Intuitive elements in the diagnostic process seem to play an important role in diagnostic reasoning (16). For example, the physician's feeling that “there was something wrong” turned out to predict serious infections in children (17). Personal preferences play an important role not only in taking medical history but also for assessing diagnostic thresholds and how to interpret them (18), probably as a function of experience and specialization. In this case, history taking in VaD may be less evidence-based than proposed.

In the current study, we validated a set of questions proposed by neurootological experts by a data-driven approach and hypothesized that a limited number of key characteristics will facilitate the differentiation of the most prevalent vestibular disorders with sufficient validity. To identify these key characteristics, several different statistical approaches are possible. In a previous study, we could show that machine learning methods may display good prediction accuracy but do not yield information about the causal pathways leading to good prediction (19). However, there are methods such as classification and regression trees (20) and their more recently developed methodological refinements (21) to rank characteristics according to their importance.

The aim of this study is to identify a set of key variables that are based on both expert experience and empirical knowledge, can be easily collected in clinical practice, and can be used as indicators for correct diagnosis of the most common vestibular disorders.

## Materials and Methods

To assemble an *a priori* collection of variables that are meaningful according to expert opinion, we first conducted a worldwide online survey among experts in the field about relevant themes and aspects of history taking in vestibular disease. We then used qualitative content analysis to clarify and structure these aspects. Thirdly, resulting contents were linked to variables from a specialized clinical patient registry that contained verified diagnoses according to current diagnostic guidelines as a gold standard. Fourthly, we used classification trees to analyze if these variables with apparent face validity also had statistical predictive validity.

### Online Survey for Expert Opinions

#### Data Collection

This survey was conducted as an anonymous online survey in 2018. The participants were recruited from members of the Bárány Society, an international society for experts committed to vestibular disorders, and members of the DizzyNet, a European network initiative for vertigo and balance research (22). The online questionnaire was created with SoSci Survey (23) and made available to the participants on “www.soscisurvey.com.” All experts were contacted by e-mail and provided with detailed information about the study.

##### Measures

The experts were asked to specify a limited number of questions that would be most salient and relevant during history taking to establish a preliminary diagnosis, e.g., to differentiate vestibular from non-vestibular or peripheral from central etiologies of VaD, along with any response options that would be indicative of a specific diagnosis. In addition to this, the respondents provided information on their institution, country, clinical specialization, and personal experience in the field.

### Structuring Expert Opinions

Structured content analysis (24–26) was used to develop categories from the text passages provided by the experts. Two researchers (EG and RS) read the text and, independently from each other, identified “meaning units,” i.e., distinct meaningful and manageable units. We then organized these units into a taxonomy consisting of main categories and subcategories hierarchically nested within main categories. To give an example, “associated symptoms” was defined as a main category, with “visual symptoms, oscillopsia” and “headache” being subcategories, respectively. The results of the two independent analyses were then synthesized. In case of differences, the final structure was decided on by discussion and consensus. MaxQDA® 2020 was used to support the content analysis and to assign weights to frequently used codes and priorities for interpretation (27).

### Linking of Expert Opinion to Registry

#### Database

DizzyReg is an ongoing prospective clinical patient registry that collects information currently stored in electronic health records and medical discharge letters to create a comprehensive clinical database of patient characteristics, symptoms, diagnostic procedures, diagnosis, therapy, and outcomes in patients with VaD (28). Routinely, the patients also report quality of life and functioning in a few standardized questionnaires (2, 29–32). Adult patients are included if they presented at the German Center for Vertigo and Balance Disorders (DSGZ), a tertiary reference unit for outpatients at the Hospital of Ludwig-Maximilians-Universität, Munich, and provided written informed consent. Recruitment into the registry commenced in December 2015. Data protection clearance and institutional review board approval has been obtained (no. 414-15).

#### Linking Procedure

We chose those variables from DizzyReg that would correspond most closely to the categories from our content analysis described above, e.g., the subcategory “duration of attacks” would fit to the corresponding variable “If you had vertigo, how long did it last? Less than 20 min; up to 20 min; 20 min to 1 h; several hours; more than 12 h; several days.” If more than one variable could be linked, we chose the variable which was most accurately measurable and which had the smallest number of missing values.

### Prediction of VaD Diagnoses

#### Ascertainment of Diagnoses

Diagnoses in the DizzyReg are based on a complete neurootological workup carried out by experienced neurootologists of the DSGZ and which conform to current guidelines (33–42). The neurootological examination includes a comprehensive battery of bedside tests, audiologic and vestibular function tests, and, if necessary, further imaging (e.g., cranial MRI) or consultation with other medical specialties, e.g., otorhinolaryngology, neurology, psychiatry, or ophthalmology. For this study, we included the seven most frequent diagnoses at the DSGZ (28, 43): benign paroxysmal positional vertigo (BPPV), functional dizziness (FD), Menière's disease (MD), vestibular paroxysmia (VP), unilateral vestibulopathy (UVP), bilateral vestibulopathy (BVP), and vestibular migraine (VM). There were no patients with both definite MD and VM in our data set.

#### Statistical Analyses

For data description, we used mean values and standard deviation for continuous variables and absolute and relative frequencies for categorical variables. In DizzyReg, missing values are routinely replaced by a neutral value, reflecting the practice that tests will not be applied if they are not needed or that symptom status are not reported if not indicated, i.e., the result is expected to be neutral.

#### Classification and Regression Trees

Classification and regression trees (CART) have the main advantage to yield a visually attractive tree structure that mimics human decision making and is easy to interpret (20). In brief, the CART procedure starts by splitting the entire data set with all individuals into smaller subsets that are more homogenous regarding a defined outcome in the current study “diagnosis.” This splitting process is visualized by an upside-down tree structure, with each node in the tree representing a variable and each branch representing a split of the data.

For example, a first split assigns all individuals reporting headache to the left subnode and all individuals not reporting headache to the right subnode. The left subnode would then contain a higher percentage of persons with VM than the right subnode. However, the left subnode would also contain persons who reported headache but were not diagnosed with VM. Each of these subnodes is subsequently split again until each branch terminates in an end node, which is maximally homogeneous regarding diagnosis. Each end node will be assigned with the class, which occurs most often in it. A patient is allocated according to his/her individual characteristics to a certain end node and subsequently classified with the diagnosis assigned to the class of this end node.

Without any restrictions, the final tree would grow until it perfectly classifies each individual in the data set but would perform poorly to classify new individuals, i.e., it overfits. Thus, the final tree needs to be shrunk (“pruned”) (20) to gain this external validity. We applied cost-complexity pruning that yields a trade-off between the complexity of the tree and its fit to the data. To get an unbiased estimate of this fit, we applied *k*-fold cross-validation (CV). CV assesses how a classification method will generalize to an independent data set by using out-of-sample estimates. The *k*-fold CV partitions the data set into *k* distinct subsets, trains the model on *k*-1 of the subsets, and estimates the test error on the remaining one. This will be repeated *k* times, with each subset acting once to assess the performance. The final fit is calculated as the average over the *k* estimates. Following common recommendations, *k* was set to 10 (44).

Some diagnoses were more frequent than others, resulting in imbalanced data. Imbalanced data may bias the tree toward the majority class, i.e., the final tree assigns individuals predominantly to the most frequent diagnosis. To avoid this, cases were weighted with the inverse of their class frequency (45), and class assignment of the end nodes was based on these weighted cases. Thus, cases with less frequent diagnoses were weighted higher than cases with more frequent diagnoses, for example, patients diagnosed with functional dizziness were weighted by 0.44 (1,066 divided by 7 × 346) and patients with unilateral vestibulopathy by 1.34 (1,066 divided by 7 × 114).

#### Estimating Variable Importance

In contrast to standard regression methods, a classification tree does not indicate which of the variables contributed most to the result, i.e., which questions will be most relevant for the diagnostic decision. To estimate variable relevance, we applied random forest classification (46), which yields estimates of variable importance values (47–49).

To assess variable importance, we applied an importance measure based on permutation (46). Here the mean decreases in accuracy, i.e., the proportion of correctly classified patients, for each variable is assessed by randomly permuting the values of this variable and measuring the decrease in accuracy due to this permutation. This permutation importance does not measure the full effect on prediction of a variable because other variables could act as surrogates. Recent developments also suggest other concrete importance parameters, among others the number of time a variable formed the root of a tree (50). In the current study, we report both the prediction accuracy based on permutation and the number of times a variable was used to split the root node.

Statistical significance was set at a two-tailed 5% level. R 3.6.1 was used for descriptive analyses (51) and the machine learning library scikit-learn (52) for learning and pruning the tree. Variable importance was assessed with the “RandomForestExplainer” package in R (50). Visualization of the trained tree was obtained using the open-source python library dtreeviz (53).

#### Diagnostic Properties

Overall accuracy was estimated as the number of correctly classified patients divided by the total number of included patients. Thus, a patient was correctly classified if the assigned class of the end node that the patient belongs to matches the final diagnosis made at the DSGZ and incorrectly if otherwise. To judge the quality of the classification for each VaD syndrome, we reported sensitivity (SEN), specificity (SPEC), positive predictive value (PPV), and negative predictive value (NPV). As these measures are only defined for binary classification and not for multi-class classification, we reduced the classification problem by the “one vs. all” approach exclusively for this calculation.

## Results

In total, 21 experts from 16 different countries took part in the online survey. The experts worked in a total of 19 centers treating an average of 1,000 patients per year (median: 700, range: 40–4,000). The participants reported an average of 23 years of clinical experience (median: 25, range: 4–42). A total of 152 different statements were reported.

Content analysis yielded nine main categories and 39 subcategories, which are shown in Table 1.

**Table 1:** Main and subcategories identified from the expert survey.

**Main category**	**Subcategory**
Description of attacks/episodes	Duration of attacks
	Episodic/continuous
	Strength of attacks
	Evolution of attacks
	Frequency of attacks
	Type of vertigo
Associated symptoms	Aural symptoms
	Headache
	Visual symptoms, oscillopsia
	Photophobia, phonophobia
	Gait/balance unsteadiness
	Psychological symptoms
	Nausea/vomiting
	Neurological symptoms
	Autonomic symptoms
	Cervical tension/pain
Medication	
Effect on daily life	
Comorbidities	Musculoskeletal
	Diabetes
	Autoimmune disease
	Psychiatric (anxiety, depression)
	Neurological
	Cardiovascular disease
Trigger	Alcohol
	Noise
	Movement
	Pressure change (air pressure, valsalva)
	Specific situation
	Trauma
	Stress/lack of sleep
	Current herpes viral infection
	Changing body position
Family history	Comorbidities
	Vertigo
	Hearing loss
	Migraine
Duration of disease (first–last episode)	Last episode
	Age of onset
Mitigating factors	

A total of 98 variables contained in the DizzyReg could be linked to one of the categories. Ten categories were not represented in the registry, e.g., alcohol or pressure changes as a trigger. A complete description of the linking results can be found in the electronic appendix (Supplementary Table 1).

We included a total of 1,066 patients with a mean age of 51.1 years (standard deviation, SD = 15.3), 56% of whom were female. Functional dizziness was the most frequent diagnosis (32.5%), followed by vestibular migraine (20.2%) and Menière's disease (13.3%). A total of 47% of patients had vertigo or dizziness for <2 years (for more details, see Table 2).

**Table 2:** Description of the study sample for the seven different diagnoses.

**Variable**	**Levels**	**All**	**FD**	**VM**	**MD**	**BPPV**	**UVP**	**BVP**	**VP**
Sample size	–	1,066	346	215	142	134	114	66	49
Gender	Female	602 (56%)	178 (51%)	145 (67%)	78 (55%)	88 (66%)	66 (58%)	27 (41%)	20 (41%)
Age	–	51.06	47.19	44.48	53.42	57.04	56.95	64.97	51.59
		(SD = 15.29)	(SD = 14.51)	(SD = 13.95)	(SD = 13.3)	(SD = 12.06)	(SD = 15.01)	(SD = 16.96)	(SD = 14.16)
Falls last 12 months	Yes	288 (27%)	74 (21%)	53 (25%)	38 (27%)	41 (31%)	36 (32%)	26 (39%)	20 (41%)
Time since first onset	<3 months	191 (18%)	69 (20%)	48 (22%)	20 (14%)	18 (13%)	25 (22%)	8 (12%)	3 (6%)
	3 months to 2 years	314 (29%)	103 (30%)	47 (22%)	39 (27%)	45 (34%)	48 (42%)	22 (33%)	10 (20%)
	2–5 years	264 (25%)	88 (25%)	54 (25%)	32 (23%)	31 (23%)	25 (22%)	15 (23%)	19 (39%)
	5–10 years	160 (15%)	49 (14%)	33 (15%)	23 (16%)	23 (17%)	11 (10%)	11 (17%)	10 (20%)
	More than 10 years	137 (13%)	37 (11%)	33 (15%)	28 (20%)	17 (13%)	5 (4%)	10 (15%)	7 (14%)
Vertigo	Yes	574 (54%)	121 (35%)	131 (61%)	113 (80%)	102 (76%)	60 (53%)	20 (30%)	27 (55%)
Postural imbalance	Yes	609 (57%)	216 (62%)	113 (53%)	74 (52%)	64 (48%)	65 (57%)	44 (67%)	33 (67%)
Dizziness	Yes	555 (52%)	223 (64%)	110 (51%)	66 (46%)	58 (43%)	55 (48%)	22 (33%)	21 (43%)

*FD, functional dizziness; VM, vestibular migraine; MD, Menière's disease; BPPV, benign paroxysmal positional vertigo; UVP, unilateral vestibulopathy; BVP, bilateral vestibulopathy; VP, vestibular paroxysmia*.

**Figure 1:**
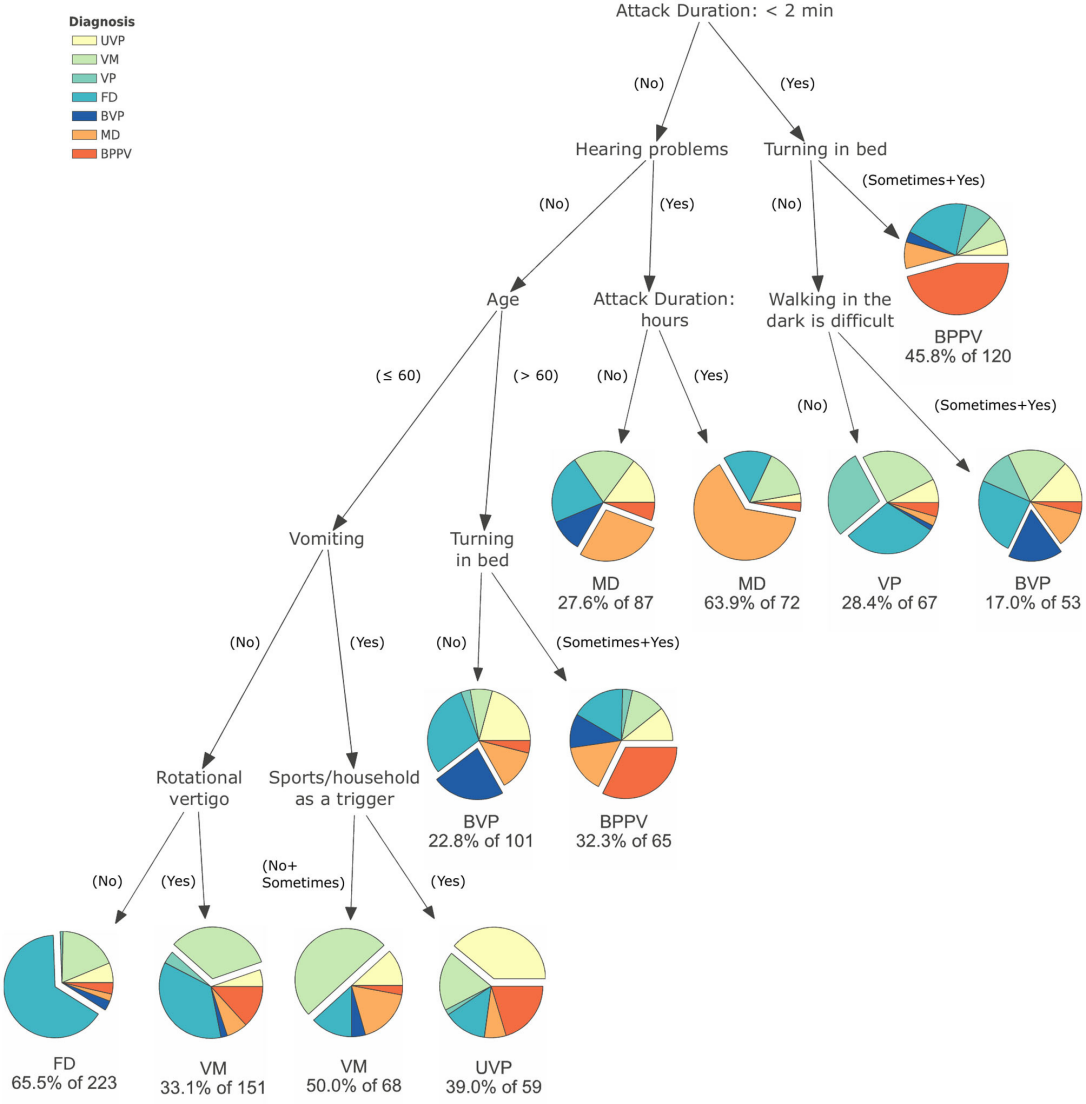
Result of the classification and regression trees to distinguish between functional dizziness, vestibular migraine, Menière's disease, benign paroxysmal positional vertigo, unilateral vestibulopathy, bilateral vestibulopathy, and vestibular paroxysmia.

Eight variables were found to be indicative for vertigo and dizziness diagnoses: attack duration, rotational vertigo, hearing problems, turning in bed as a trigger, doing sport or heavy household chores as a trigger, age, having problems with walking in the dark, and vomiting. The resulting tree is shown in Figure 1.

To give an example for interpretation: two paths in the tree could identify 56.7% of patients with BPPV correctly. In the first path, a short attack duration (<2 min) and turning in bed as a trigger lead to a correct classification of 55 patients with BPPV. In the second path, a longer attack duration, no hearing problems, age >60, and turning in bed as a trigger lead to 21 patients being correctly classified. In summary, of the 134 patients with BPPV, 76 (56.7%) were assigned to the correct classification of BPPV. The overall accuracy of the algorithm for all diagnoses as a multi-class problem was 42.2%. Further details of the diagnostic properties can be found in Table 3.

**Table 3:** Comparison of the classification of the classification and regression trees algorithm with the diagnosis made at the German Center for Vertigo and Balance Disorders (DSGZ) for functional dizziness (FD), vestibular migraine (VM), Menière's disease (MD), benign paroxysmal positional vertigo (BPPV), unilateral vestibulopathy (UVP), bilateral vestibulopathy (BVP), and vestibular paroxysmia (VP).

		**Diagnosis by DSGZ**							**Diagnostic parameters**			
		**FD**	**VM**	**MD**	**BPPV**	**UVP**	**BVP**	**VP**	**SENS (%)**	**SPEC (%)**	**PPV (%)**	**NPV (%)**
Diagnosis by classification algorithm	FD	146	41	5	8	14	7	2	42.2	57.8	65.5	34.5
	VM	63	84	22	22	16	6	6	39.1	60.9	38.4	61.6
	MD	30	28	70	7	15	9	0	49.3	50.7	44.0	56.0
	BPPV	36	17	20	76	13	11	12	56.7	43.3	41.1	58.9
	UVP	8	11	4	12	23	0	1	20.2	79.8	39.0	61.0
	BVP	43	17	19	6	28	32	9	48.5	51.5	20.8	79.2
	VP	20	17	2	3	5	1	19	38.8	61.2	28.4	71.6

*Performance is described by SENS, sensitivity; SPEC, specificity; PPV, positive predictive value, and NPV, negative predictive value*.

The variable importance of the 20 variables that contributed most to the classification was determined by random forest analysis and is shown in Table 4. The variable with the most influence on the performance of the algorithm was vomiting, followed by age and hearing problems.

**Table 4:** Variable importance of the 20 most relevant variables to differentiate between the seven different vestibular diagnoses (functional dizziness, vestibular migraine, Menière's disease, benign paroxysmal positional vertigo, unilateral vestibulopathy, bilateral vestibulopathy, and vestibular paroxysmia).

**Variables**	**Mean decrease in accuracy**	**Root node^#^**
Vomiting	1.41	744
Age	1.02	978
Hearing problems	0.93	682
Turning in bed as a trigger	0.92	343
Attack duration: <2 min	0.76	1,105
Rotational vertigo	0.67	338
Getting in and out of bed is difficult	0.63	138
Attack duration: hours	0.61	446
Nausea	0.57	481
Positional maneuver as a trigger	0.38	223
Ear pressure	0.29	321
Walking in the dark is difficult	0.27	780
Walking on sidewalks is difficult	0.24	504
Ear noise	0.20	72
Attack duration: several days	0.16	491
Provocational nystagmus	0.16	46
Gait disturbance	0.16	268
Bending over as a trigger	0.16	62
Eye movement disorder	0.14	52
Headache	0.12	31

*The estimation of importance measures was based on random forests with 10,000 trees. The mean decrease in accuracy was based on permutation in importance*.

# *Root node indicates how often a variable was used to split the root node (higher frequencies indicate higher relevance for the classification)*.

## Discussion

This study was able to identify a set of eight key questions that can help to differentiate seven common vestibular diagnoses. The key questions comprised attack duration, rotational vertigo, hearing problems, turning in bed as a trigger, doing sport or heavy household chores as a trigger, age, having problems with walking in the dark, and vomiting.

The negative predictive values were higher than the positive predictive values, indicating that it was mostly easier to exclude a diagnosis than to confirm it.

Using expert opinion and a statistical classification approach, we arrived at combinations of symptoms with high face validity. Positive predictive value in our study was highest for functional dizziness. This is not surprising because FD is characterized by the combination of typical symptoms and the absence of others (38). Thus, the sequence of longer attack duration, no hearing problems, younger age, no vegetative symptoms, and a presentation as dizziness rather than rotational vertigo indicated FD, which is in line with the approach presented by Dieterich et al. (54, 55). On the other hand, FD was also frequently present in other nodes (between 13 and 29%). This finding may be explained by the relatively high prevalence of FD in this sample. Furthermore, patients with FD report a multitude of uncharacteristic symptoms fluctuating in time and intensity, triggered by various situations (55). Furthermore, FD often manifests as a comorbidity to or consequence of different organic vestibular disorders, most commonly VM, BPPV, and MD (56).

BPPV was likewise characterized in our study by a short duration of attacks and the movement of body and head in the horizontal plane, which is in line with the typical clinical presentation (43, 57). These two questions identified more than half of BPPV patients. Head movement while turning in bed was also indicative for an older group of patients with BPPV who had longer attacks. This finding also aligns with literature (58).

In addition, the characterization of MD, BVP, and UVP in our study is in accordance with the clinical key features described previously (39, 59, 60). In our study, MD patients had a longer attack duration and hearing loss. BVP was characterized by symptoms aggravating in darkness, permanent dizziness, and higher age. Increase of symptoms in darkness and older age are highly characteristic for BVP (61). UVP characteristics were a longer duration of symptoms, lack of hearing loss, younger age (<60 years), vomiting, and aggravation by sports or heavy household chores. Although UVP can occur in all age groups and is thus not considered to be typical for younger adults, a peak between the ages of 30 and 50 was suggested (62).

Vestibular migraine was difficult to classify in our study. The clearest differentiation was age, which is confirmed by the finding that VM typically manifests in younger adults (63) without typical triggers (34). Interestingly, we were not able to confirm headache as a typical feature of VM. It has been shown that about 30% of patients with VM do not report headache associated to vertigo attacks (64), and <50% of patients report the simultaneous presence of headache and vertigo during attacks (65).

A specific objective of the present study was to train a machine learning model for classification that is transparent, easy to use in daily clinical practice, and easy to understand. In the past, several different approaches have been used to help classify the underlying pathologies of vertigo and dizziness. A complex method like deep neural networks (DNN), which was applied to vestibular disorders with promising results (19, 66), is difficult to directly transfer to a real-world clinical setting since DNN does not provide transparency on how the classification decision was made. Similarly, applying support vector machine (SVM) is accompanied by a lack of procedural transparency but comes with a high power regarding accuracy. A study on classifying unilateral vestibulopathy using SVM yielded an accuracy of 76% (67). Another study was able to differentiate the peripheral and central causes of acute vestibular disorders with a high accuracy using modern machine learning methods (68). Recent research aimed to alleviate the drawback of procedural opacity and develop explainable artificial intelligence (69), but their work was based on pattern recognition and has not been applied to data sets with numeric, ordinal, or categorical data yet. Thus, further research is required to identify if such an approach might be appropriate for a clinical decision-making system in specialized areas like vestibular disorders.

Studies using transparent learning methods, like CART, are scarce. A study from 2000 also applied classification trees for differentiation of vestibular disorders (70). They identified hearing loss, duration of the disease, frequency of attacks, severity of rotational vertigo, onset and type of hearing loss, and occurrence of head injury at onset of vertigo as important variables for diagnostic classification. These variables are similar to the ones presented here, although some variables were not surveyed in the present study (e.g., association to trauma). Another study used boosted decision trees to identify two different feature sets, one for general practitioners and one for experts (71). All these studies reported a higher accuracy than our study, as they used a one-vs.-all classification approach, which results in better accuracy but is less precise than our approach. To put this into context, a one-vs.-all approach has an implicit minimal accuracy of 50% as the classification problem is reduced to a dichotomous choice. In contrast to this, our algorithm aims at distinguishing between seven different vertigo syndromes simultaneously, yielding a minimal accuracy of 1/7 = 14%. Thus, the overall accuracy of 42.2% is a notable improvement.

### Limitations

In our study, we used data from a patient registry of a tertiary referral center for balance disorders, which is not representative for patients presenting with vertigo and dizziness in primary or secondary care. Patients visiting specialized units are usually a selection of severe or chronic patients with a long history of disease or unsuccessful therapy. This may explain the low accuracy of our findings. However, this registry is one of the largest data collections of information on vestibular disorders, including rare forms, and belongs to one of the most comprehensive and valid sources for clinical phenotyping. In addition, there are patients with overlapping syndromes (e.g., VM and MD). This overlap may pose a challenge for the current diagnostic approach as these patients may present with a set of symptoms not characteristic for the assigned diagnosis. Furthermore, certain syndromes occurring in the emergency setting, like vestibular TIA or stroke, or other rare syndromes, like vestibular schwannoma, are not sufficiently represented in the patient registry. To be applied in a real-world setting, these vestibular syndromes should be incorporated in the algorithm, for example, by including expert knowledge or data sets from emergency departments. To further improve the diagnostic algorithm for the classification of common vestibular disorders, results from basic clinical vestibular testing (such as the clinical head impulse test or positioning maneuvers) and a modification of symptomatic categories need to be incorporated into the model.

There are several shortcomings of CART as opposed to other methods. Firstly, trees are not very robust to small changes in the data, i.e., a small change can result into a different tree. Furthermore, CART cannot handle non-random missing values in an adequate way. A common approach to handle missing values is based on surrogate splits, which cannot be applied here, as the best surrogate candidates have the same cause of missingness, e.g., the decision of the physician that a certain measurement is not necessary for diagnosis (72). Secondly, trees cannot compete with complex ensemble methods in terms of prediction accuracy alone. A study using real and simulated data sets showed that the accuracy of the best single-tree algorithm is on average about 10% less than that of a tree ensemble (21).

However, we are confident that, for the purpose of the current study, CART represents the most transparent method to develop an algorithm for diagnosing vestibular disorders. The main advantage of tree-based models is that they mirror human decision-making. Thus, the identified tree can act as a blueprint for taking and structuring patient records.

### Conclusion

The presented algorithm used a transparent and easily applicable approach for categorizing different common vestibular syndromes based on eight key questions. It may be helpful for the initial triage of patients but needs to be followed by a basic clinical exam of vestibular and ocular motor functions to improve the accuracy of the diagnostic classification. To evaluate if the identified algorithm might be a basis for a simple-to-use algorithm in a primary care setting, further studies are required.

## Data Availability Statement

The data analyzed in this study is subject to the following licenses/restrictions: an application form has to be sent to the Scientific Committee of the German Center for Vertigo and Balance Disorders. Requests to access these datasets should be directed to ralf.strobl@med.uni-muenchen.de.

## Ethics Statement

The studies involving human participants were reviewed and approved by Ethic committee of the medical faculty, LMU Munich, Munich, Germany. The patients/participants provided their written informed consent to participate in this study.

## Author Contributions

RS contributed to the drafting/revising of the manuscript for content, including writing, study concept/design, interpretation of data, and acquisition of data. MG contributed to statistical analysis and revision of the manuscript for content, including writing, and study concept. AZ contributed to the interpretation of data and revision of the manuscript for content. DH contributed to the development of the concept and revision of the manuscript for content. FF contributed to the interpretation of data, revision of the manuscript for content, and medical writing. EG contributed to the drafting/revision the manuscript for content, including medical writing and study concept/design. All authors contributed to the article and approved the submitted version.

## Conflict of Interest

The authors declare that the research was conducted in the absence of any commercial or financial relationships that could be construed as a potential conflict of interest.
