# Supplementary material for: A Set of Eight Key Questions Helps to Classify Common Vestibular Disorders—Results From the DizzyReg Patient Registry
Source: Front Neurol. 2021 Apr 29;12:670944. doi: 10.3389/fneur.2021.670944 (PMC8116658; doi:10.3389/fneur.2021.670944)
Supplement: Supplementary file 1 [file Table_1.DOCX]

**eTable 1** Variables in the database and the respective categories of the taxonomy as derived from the content analysis.

| **Main category** | **Subcategory** | **Variable** |
| --- | --- | --- |
| 1 Description of attacks/episodes | Duration of attacks | Attack duration |
|  | Episodic/Continuous | Permanent vertigo |
|  | Strength of attacks | - |
|  | Evolution of attacks | - |
|  | Frequency of attacks | Permanent vertigo |
|  | Type of vertigo (Dizziness, Rotational,  Being in boat, Gait or Balance problems) | Dizziness, Postural imbalance, Vertigo, Walking problems, Standing problems |
| 2 Accompanying symptoms | Aural symptoms | Hearing problems, Ear pressure, Ear noise |
|  | Headache | Headache, Head pressure |
|  | Visual symptoms, Oscillopsia | Blurring pictures, Double vision, Eye movement, Impaired vision, Blurred vision, Spontaneous nystagmus, Positional nystagmus |
|  | Photo-, phonophobia | Light sensitivity |
|  | Gait/balance unsteadiness | Walk in the dark, Walking problems, Falls |
|  | Psychological symptoms | Feeling frustrated, Depression |
|  | Nausea/Vomiting | Nausea, Vomiting |
|  | Neurological symptoms | Sensory deficits in arms, legs, or face, Fine motor skills, Blind going, Line going, Toe going, Heel going, Small steps, Broad basis, Dysarthria, Orientation to time, Orientation to place, Orientation to person, Orientation to situation |
|  | Autonomic symptoms | Dizziness |
|  | Cervical tension/pain | Neck pain |
| 3 Medication |  | Medication |
| 4 Effect on daily life |  | Getting in/out of bed, Reading, Homework |
| 5 Comorbidities | Musculoskeletal | Joint disease |
|  | Diabetes | Diabetes |
|  | Autoimmune disease | Multiple sclerosis |
|  | Psychiatric (Anxiety, Depression) | Anxiety, Depression |
|  | Neurological | Neurological disease |
|  | Cardiovascular disease | Heart failure, Heart disorder, Heart rhythm, Blood pressure |
| 6 Trigger | Alcohol | - |
|  | Aural triggers | Noises |
|  | Movement | Going sidewalk, Supermarket, Sports/Household |
|  | Pressure change | - |
|  | Specific situation | Crowds, Design of buildings, Quick movements |
|  | Trauma | - |
|  | Stress/lack of sleep | Problems falling asleep, Problem to sleep through |
|  | Herpes | Herpes |
|  | Changing body position | Looking up, Bending over, Head movement, Turning in bed |
| 7 Family history | Comorbidities | - |
|  | Vertigo | - |
|  | Hearing loss | - |
|  | Migraine | - |
| 8 Duration of disease (first - last episode) | Last episode | - |
|  | Age of onset | Time since first onset, Age |
| 9 Mitigating Factors |  | - |
